# DPP7 Promotes Colorectal Cancer Progression Through GPX4‐Dependent Suppression of Disulfidptosis and Immune Evasion

**DOI:** 10.1111/jcmm.70660

**Published:** 2025-06-27

**Authors:** Ruibing Li, Xinyou Wang, Jun Liu, Zeyu Cai, Zhu Li, Qiang Tao, Chong Wang

**Affiliations:** ^1^ Key Laboratory of Biological Targeting Diagnosis, Therapy and Rehabilitation of Guangdong Higher Education Institutes The Fifth Affiliated Hospital, Guangzhou Medical University Guangzhou Guangdong People's Republic of China; ^2^ Guangdong Provincial Key Laboratory of Colorectal and Pelvic Floor Diseases The Sixth Affiliated Hospital, Sun Yat‐Sen University People's Republic of China; ^3^ Biomedical Innovation Center The Sixth Affiliated Hospital, Sun Yat‐Sen University People's Republic of China; ^4^ Department of General Surgery (Colorectal Surgery), The Sixth Affiliated Hospital Sun Yat‐Sen University People's Republic of China; ^5^ Department of General Surgery (Stomach Surgery), The Sixth Affiliated Hospital Sun Yat‐Sen University People's Republic of China; ^6^ The First Affiliated Hospital of Anhui University of Chinese Medicine Hefei People's Republic of China; ^7^ The Sixth Affiliated Hospital South China University of Technology Foshan Guangdong People's Republic of China; ^8^ Nanhai District People's Hospital of Foshan Foshan Guangdong People's Republic of China; ^9^ Department of Hepatobiliary and Pancreatic Surgery, the Eighth Afffliated Hospital Sun Yat‐Sen University Shenzhen People's Republic of China; ^10^ Department of General Surgery The Fifth Affiliated Hospital of Guangzhou Medical University Guangzhou Guangdong People's Republic of China

**Keywords:** colorectal cancer, disulfidptosis, DPP7, GPX4, immune evasion

## Abstract

Colorectal cancer (CRC) remains a leading cause of cancer‐related mortality worldwide, highlighting the need to identify novel mechanisms driving tumour progression. In this study, we demonstrate that dipeptidyl peptidase 7 (DPP7) high expression correlates with poor prognosis in CRC patients. Functional analyses revealed that DPP7 promotes CRC cell proliferation and inhibits apoptosis, while its depletion enhances natural killer (NK) cell‐mediated cytotoxicity against tumour cells. Mechanistically, we identified a previously unknown role of DPP7 in suppressing disulfidptosis, a novel form of regulated cell death characterised by excessive formation of intracellular disulfide bonds. DPP7 overexpression protected CRC cells from glucose deprivation‐induced disulfidptosis, as evidenced by reduced disulfide bond formation in cytoskeletal proteins including drebrin, FLNA and FLNB. Furthermore, we discovered that DPP7 physically interacts with glutathione peroxidase 4 (GPX4), a key regulator of cellular redox homeostasis, and stabilises GPX4 protein without affecting its mRNA expression. GPX4 restoration in DPP7‐depletion cells reversed the enhanced sensitivity to both disulfidptosis and NK cell‐mediated killing, while GPX4 depletion abolished the protective effect of DPP7 overexpression. Our findings unveil a novel DPP7‐GPX4 axis in regulating disulfidptosis and immune evasion in colorectal cancer, providing potential therapeutic targets for CRC treatment. Targeting this pathway may simultaneously inhibit tumour cell survival mechanisms and enhance immune‐mediated tumour elimination.

## 1 Introduction

Colorectal cancer (CRC) remains one of the most prevalent and lethal malignancies worldwide, ranking as the third most common cancer and the second leading cause of cancer‐related deaths globally [1]. Despite notable progress in screening, diagnosis and therapeutic approaches, the prognosis for patients with advanced colorectal cancer (CRC) remains unfavourable. This highlights the pressing need to elucidate novel molecular mechanisms underlying CRC progression and to identify potential therapeutic targets. Dipeptidyl peptidase 7 (DPP7), also known as quiescent cell proline dipeptidase (QPP), is a post‐proline cleaving aminopeptidase belonging to the S28 family of serine proteases [2]. Unlike its well‐characterised family member DPP4, which has been extensively studied in cancer biology, the role of DPP7 in tumorigenesis remains largely unexplored. Recent studies have implicated DPP7 in various cellular processes, including protein turnover, immune regulation and cellular homeostasis [3, 4, 5, 6]. However, its precise role in the development and progression of colorectal cancer has not yet been fully explored.

The survival of cancer cells under adverse conditions, such as nutrient deprivation, hypoxia and oxidative stress, is critical for tumour progression and metastasis. Emerging evidence suggests that cancer cells develop sophisticated mechanisms to adapt to these stressors and evade cell death [7, 8]. Disulfidptosis, a recently identified form of regulated cell death characterised by excessive formation of intracellular disulfide bonds leading to protein aggregation and cellular dysfunction, has been implicated in various pathological conditions, including cancer [9]. The redox homeostasis, particularly the balance between reactive oxygen species (ROS) and antioxidant systems, plays a critical role in regulating disulfidptosis [10]. However, the molecular mechanisms regulating susceptibility to disulfidptosis in colorectal cancer are still not well understood.

Glutathione peroxidase 4 (GPX4), a key regulator of lipid peroxidation and redox homeostasis, has emerged as a central player in ferroptosis, another form of regulated cell death [11]. GPX4 catalyses the reduction of lipid hydroperoxides to their corresponding alcohols, thereby preventing the accumulation of toxic lipid peroxides. Recent studies have highlighted the importance of GPX4 in cancer cell survival, drug resistance and tumour progression [12, 13]. However, the potential interplay between GPX4 and disulfidptosis, especially within the context of colorectal cancer, still needs to be clarified.

Immune evasion is a hallmark of cancer that contributes significantly to tumour progression and therapy resistance [14, 15, 16]. Natural killer (NK) cells, as key components of the innate immune system, play a crucial role in tumour immunosurveillance through direct cytotoxicity against malignant cells and production of pro‐inflammatory cytokines [17]. The sensitivity of tumour cells to NK cell‐mediated killing is regulated by a complex interplay of activating and inhibitory signals, as well as tumour‐intrinsic factors that modulate NK cell recognition and function [18]. Understanding the molecular mechanisms behind tumour immune evasion from NK cells could offer valuable insights for developing effective immunotherapeutic strategies.

In this study, we aimed to investigate the role of DPP7 in colorectal cancer progression and its potential involvement in regulating disulfidptosis and immune evasion. We observed that high expression of DPP7 is associated with poor patient prognosis. Functional studies revealed that DPP7 promotes CRC cell proliferation and survival while suppressing NK cell‐mediated cytotoxicity against tumour cells. Mechanistically, we demonstrated that DPP7 interacts with GPX4 and regulates its protein stability, thereby protecting CRC cells from disulfidptosis under glucose deprivation conditions. Our findings unveil a novel DPP7‐GPX4 axis in regulating disulfidptosis and immune evasion in colorectal cancer, providing potential therapeutic targets for CRC treatment.

## 2 Materials and Methods

### 2.1 Cell Culture and Reagents

Human colorectal cancer cell lines (HCT116, SW480, RKO, HC29, DLD‐1, HT29 and SW620) and the normal colon epithelial cell line (NCM460) were obtained from the American Type Culture Collection (ATCC, Manassas, VA, USA). All cell lines were authenticated by short tandem repeat (STR) profiling and tested for mycoplasma contamination every six months. Cancer cells were cultured in DMEM (Gibco, Thermo Fisher Scientific, Waltham, MA, USA) supplemented with 10% fetal bovine serum (FBS, Gibco) and 1% penicillin/streptomycin (Gibco) at 37°C in a humidified incubator with 5% CO2. NCM460 cells were maintained in RPMI‐1640 medium (Gibco) supplemented with 10% FBS and 1% penicillin/streptomycin. For glucose deprivation experiments, cells were washed twice with PBS and cultured in glucose‐free DMEM (Gibco) supplemented with 10% dialyzed FBS (Gibco) for the indicated times. Regular DMEM containing 25 mM glucose was used as control.

### 2.2 RNA Interference and Plasmid Transfection

Small interfering RNAs (siRNAs) targeting DPP7 and GPX4, as well as their negative control siRNAs, were synthesised by GenePharma (Shanghai, China). Cells were transfected with 50 nM siRNA using Lipofectamine RNAiMAX (Invitrogen, Thermo Fisher Scientific) according to the manufacturer's instructions. For plasmid transfection, human DPP7 and GPX4 cDNAs were cloned into the pcDNA3.1(+) vector (Invitrogen). FLAG‐HA‐tagged DPP7 (FH‐DPP7), Myc‐tagged DPP7, FLAG‐HA‐tagged GPX4 (FH‐GPX4) and Myc‐tagged GPX4 were constructed by inserting the respective tag sequence at the N‐terminus of the coding region. All constructs were verified by DNA sequencing. Plasmid transfection was performed using Lipofectamine 3000 (Invitrogen) according to the manufacturer's protocol.

### 2.3 RNA Extraction and Quantitative Real‐Time PCR (qRT‐PCR)

Total RNA was extracted using TRIzol reagent (Invitrogen) according to the manufacturer's instructions. Complementary DNA (cDNA) was synthesised from 1 μg of total RNA using the PrimeScript RT Reagent Kit (Takara, Shiga, Japan). qRT‐PCR was performed using SYBR Premix Ex Taq II (Takara) on a LightCycler 480 system (Roche, Basel, Switzerland). GAPDH was used as an internal control. The primer sequences are listed as below. The relative expression levels were calculated using the 2^^‐ΔΔCt^ method.

**Table:** 

Name	Forward‐primer (5′‐3′)	Reverse‐primer (5′‐3′)
GAPDH	GGAGCGAGATCCCTCCAAAAT	GGCTGTTGTCATACTTCTCATGG
DPP7	GAAGCGTTCCGACAGATCAAG	TCAGGTCCTTCTCGTCTGACA
GPX4	GAGGCAAGACCGAAGTAAACTAC	CCGAACTGGTTACACGGGAA

### 2.4 Western Blotting

Cells were lysed in RIPA buffer (50 mM Tris–HCl, pH 7.4, 150 mM NaCl, 1% NP‐40, 0.5% sodium deoxycholate, 0.1% SDS) supplemented with protease inhibitor cocktail (Roche). Protein concentration was determined using the BCA Protein Assay Kit (Thermo Fisher Scientific). For reducing conditions, samples were mixed with 5× loading buffer containing 100 mM DTT and boiled at 95°C for 5 min. For non‐reducing conditions, protein samples were mixed with 5× loading buffer without DTT and heated at 70°C for 10 min. Equal amounts of protein (30 μg) were separated by SDS‐PAGE and transferred to PVDF membranes (Millipore, Burlington, MA, USA). The membranes were blocked with 5% non‐fat milk in TBST for 1 h at room temperature and then incubated with primary antibodies overnight at 4°C. After washing with TBST (3 × 10 min), the membranes were incubated with HRP‐conjugated secondary antibodies (1:5000, Cell Signalling Technology) for 1 h at room temperature. Protein bands were visualised using the ECL detection system (Thermo Fisher Scientific) and quantified using ImageJ software (NIH, Bethesda, MD, USA). For proliferation and apoptosis marker analysis, cells were harvested 48 h post‐transfection. The following additional primary antibodies were used: anti‐PCNA (1:1000, Cell Signalling Technology, #13110), anti‐Bcl‐2 (1:1000, Cell Signalling Technology, #15071) and anti‐Bax (1:1000, Cell Signalling Technology, #5023). Protein expression was quantified using ImageJ software and normalised to GAPDH levels.

**Table:** 

Antibodies	Source	Identifier
GAPDH Polyclonal antibody	Proteintech	#10494‐1‐AP
DPP7 Polyclonal antibody	Proteintech	#19018‐1‐AP
GPX4 Antibody	Cell Signalling Technology	#52455
Filamin A(FLNA) Antibody	Cell Signalling Technology	#4762
Filamin B (D7E4W) Rabbit mAb	Cell Signalling Technology	#12979
Drebrin Polyclonal Antibody	Thermo Fisher Scientific	#10260‐1‐AP
PCNA Antibody	Cell Signalling Technology	#13110
BCL‐2 Antibody	Cell Signalling Technology	#15071
BAX Antibody	Cell Signalling Technology	#5023

### 2.5 Co‐Immunoprecipitation

Cells were lysed in IP lysis buffer (50 mM Tris–HCl, pH 7.4, 150 mM NaCl, 1% Triton X‐100) supplemented with protease inhibitor cocktail. Cell lysates (1 mg protein) were pre‐cleared with Protein A/G PLUS‐Agarose beads (Santa Cruz Biotechnology, Dallas, TX, USA) for 1 h at 4°C with rotation. The pre‐cleared lysates were incubated with 2 μg of anti‐FLAG antibody (#F3165, Sigma‐Aldrich), anti‐GPX4 antibody (#ab125066, Abcam), or control IgG (#2729, Cell Signalling Technology) overnight at 4°C with rotation, followed by incubation with Protein A/G PLUS‐Agarose beads for 4 h at 4°C. The immunoprecipitates were washed four times with IP lysis buffer (5 min each wash), eluted by boiling in 2× loading buffer for 5 min at 95°C, and analysed by Western blotting.

### 2.6 Cell Proliferation Assay

Cell proliferation was measured using the Cell Counting Kit‐8 (CCK‐8, Dojindo, Kumamoto, Japan) according to the manufacturer's instructions. Briefly, cells were seeded in 96‐well plates at a density of 2 × 10^3^ cells per well. At the indicated time points, 10 μL of CCK‐8 solution was added to each well and incubated for 2 h at 37°C. The absorbance at 450 nm was measured using a microplate reader (BioTek, Winooski, VT, USA). Each experiment was performed in triplicate and repeated three times.

### 2.7 Colony Formation Assay

Cells were seeded in 6‐well plates at a density of 1 × 10^3^ cells per well and cultured for 14 days. The colonies were fixed with 4% paraformaldehyde for 15 min and stained with 0.1% crystal violet for 20 min. After washing with PBS, the colonies were photographed and counted using ImageJ software. Colonies containing more than 50 cells were counted.

### 2.8 Flow Cytometry Analysis of Apoptosis

Apoptosis was measured using the Annexin V‐FITC/PI Apoptosis Detection Kit (BD Biosciences, San Jose, CA, USA). Briefly, cells were harvested, washed twice with cold PBS, and resuspended in 1× binding buffer at a concentration of 1 × 10^6^ cells/ml. Then, 100 μL of cell suspension was transferred to a 5 mL culture tube, followed by the addition of 5 μL Annexin V‐FITC and 5 μL PI. After incubation for 15 min at room temperature in the dark, 400 μL of 1× binding buffer was added to each tube. The samples were analysed by flow cytometry (BD FACSCalibur, BD Biosciences) within 1 h. At least 10,000 events were collected for each sample. The data were analysed using FlowJo software (Tree Star, Ashland, OR, USA).

### 2.9 NK Cell Isolation and Cytotoxicity Assay

Human peripheral blood mononuclear cells (PBMCs) were isolated from healthy donors using Ficoll‐Paque PLUS (GE Healthcare, Chicago, IL, USA) density gradient centrifugation. NK cells were purified from PBMCs using the NK Cell Isolation Kit (Miltenyi Biotec, Bergisch Gladbach, Germany) according to the manufacturer's instructions. The purity of isolated NK cells (> 95%) was confirmed by flow cytometry. For NK cell cytotoxicity assay, target tumour cells were seeded in 96‐well plates at a density of 5 × 10^3^ cells per well. After 24 h, NK cells were added at an effector‐to‐target (E:T) ratio of 5:1 and co‐cultured for 4 h. Cytotoxicity was measured using the CytoTox 96 Non‐Radioactive Cytotoxicity Assay Kit (Promega, Madison, WI, USA) according to the manufacturer's instructions. The percentage of specific lysis was calculated as follows: % cytotoxicity = [(experimental release–effector spontaneous release–target spontaneous release)/(target maximum release–target spontaneous release)] × 100%.

### 2.10 Enzyme‐Linked Immunosorbent Assay

The levels of IFN‐γ and TNF‐α in the supernatants of NK cell‐tumour cell co‐cultures were measured using the Human IFN‐γ enzyme‐linked immunosorbent assay (ELISA) Kit and Human TNF‐α ELISA Kit (both from R&D Systems, Minneapolis, MN, USA) according to the manufacturer's instructions. Absorbance was measured at 450 nm using a microplate reader, with the wavelength correction set at 570 nm.

### 2.11 Bioinformatic Analysis

Gene expression and clinical data of colorectal adenocarcinoma (COAD) patients were obtained from The Cancer Genome Atlas (TCGA) database (https://portal.gdc.cancer.gov/). Patients were divided into high and low DPP7 expression groups based on the median expression value. Kaplan–Meier survival curves were generated, and the log‐rank test was used to assess statistical significance. Correlation analysis between DPP7 and disulfidptosis‐related genes was performed using Pearson's correlation coefficient.

### 2.12 Stress Enhancement Factor Calculation

The stress enhancement factor (SEF) was used to quantify the amplification of experimental effects under stress conditions compared to normal conditions. For glucose deprivation experiments, we calculated the SEF to assess how the effect of DPP7 overexpression or depletion was enhanced under glucose deprivation compared to normal glucose conditions. To calculate the SEF, we first measured cell viability, apoptosis, or other relevant outcomes in both control (EV or si‐NC) and experimental groups (DPP7‐overexpressing or DPP7‐depletion cells) under normal glucose (Glc+, 25 mM) and glucose‐deprived (Glc‐, 0 mM) conditions. We then calculated two effect sizes: the normal condition effect ((Experimental–Control)/Control) and the stress condition effect using the same formula but with data from glucose‐deprived conditions. The SEF was determined as the ratio of these two effects: stress condition effect divided by normal condition effect.

An SEF greater than 1 indicates that the effect of DPP7 manipulation is enhanced under glucose deprivation, equal to 1 means the effect is unchanged, and less than 1 suggests the effect is diminished under stress. For all experiments where SEF is reported, we ensured that both effects were statistically significant (*p* < 0.05). In our analysis, we considered an SEF > 5 to represent a substantial enhancement of the experimental effect under stress conditions, suggesting a specific role of the tested factor in stress response pathways. The SEF values were always interpreted alongside the raw data to avoid misinterpretation, particularly in cases where very small normal condition effects could lead to artificially inflated SEF values.

### 2.13 Statistical Analysis

Statistical analyses were performed using GraphPad Prism 8.0 software (GraphPad Software, San Diego, CA, USA). Data are presented as mean ± standard deviation (SD) of at least three independent experiments. Statistical differences between two groups were determined by Student's t‐test. One‐way ANOVA followed by Tukey's post hoc test was used for multiple comparisons. Survival analysis was performed using the Kaplan–Meier method, and the log‐rank test was used to assess statistical significance. *p* < 0.05 was considered statistically significant. *, *p* < 0.05; **, *p* < 0.01; ***, *p* < 0.001.

## 3 Results

### 3.1 DPP7 Expression Is Elevated in Colorectal Cancer and Associated With Poor Prognosis

**FIGURE 1:**
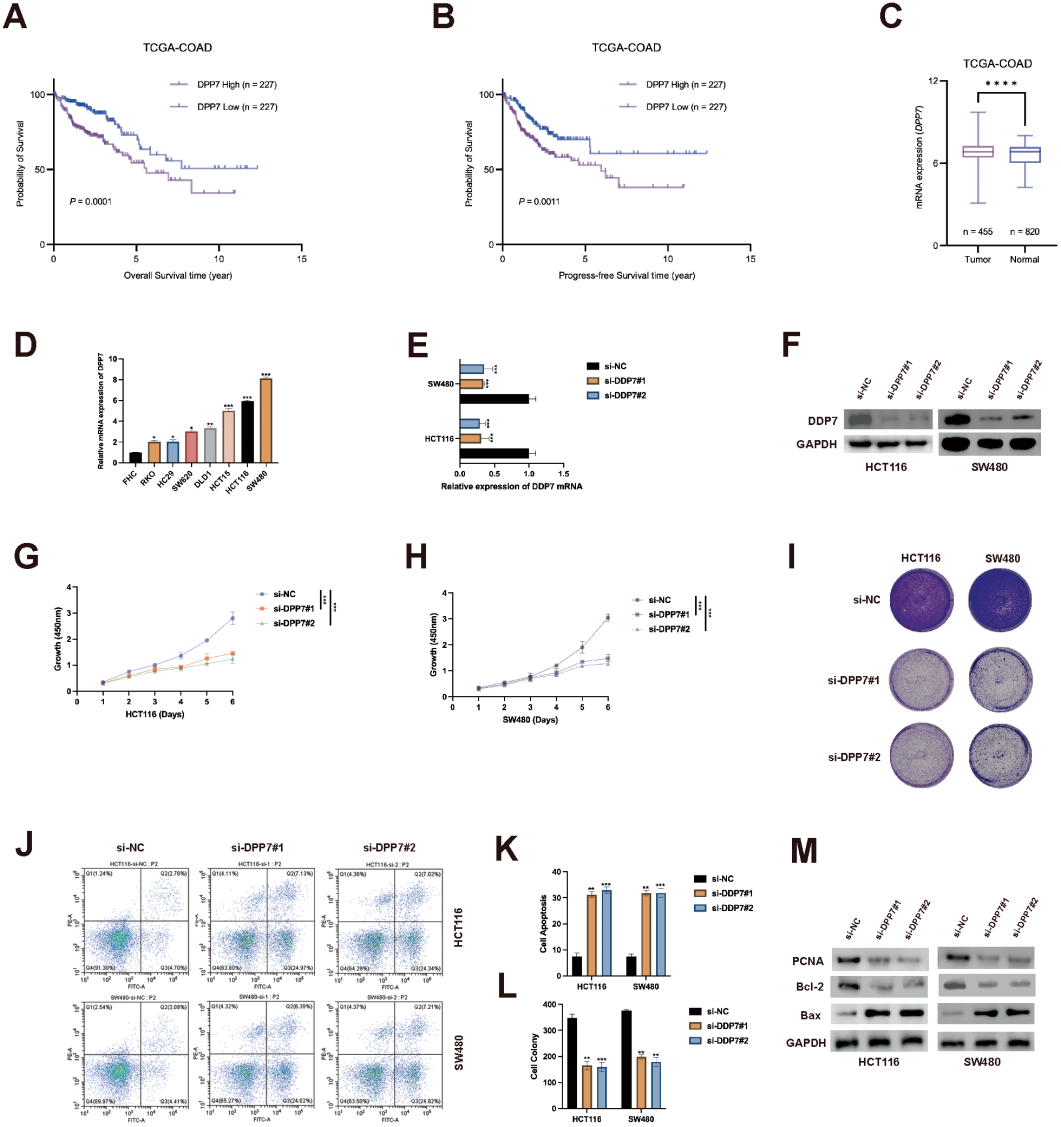
DPP7 is overexpressed in colorectal cancer and promotes tumour growth. (A) Kaplan–Meier overall survival analysis of TCGA‐COAD patients stratified by DPP7 expression levels (*n* = 227 in each group, *p* = 0.0001). (B) Kaplan–Meier progression‐free survival analysis of TCGA‐COAD patients stratified by DPP7 expression levels (*n* = 227 in each group, *p* = 0.0011). (C) DPP7 mRNA expression in tumour (*n* = 455) versus normal (*n* = 820) tissues from TCGA‐COAD dataset. (D) Relative DPP7 mRNA expression in various colorectal cancer cell lines compared to normal colon epithelial cell line (NCM). (E) Relative DPP7 mRNA expression in HCT116 and SW480 cells transfected with control siRNA (si‐NC) or DPP7‐specific siRNAs (si‐DPP7#1 and si‐DPP7#2). (F) Western blot analysis of DPP7 protein expression in HCT116 and SW480 cells transfected with indicated siRNAs. (G‐H) Cell growth curves of HCT116 (G and SW480 (H) cells transfected with indicated siRNAs measured by CCK‐8 assay. (I) Representative images of colony formation assay for HCT116 and SW480 cells transfected with indicated siRNAs. (J) Quantification of colony numbers from (I). (K) Flow cytometry analysis of apoptosis in HCT116 and SW480 cells transfected with indicated siRNAs. (L) Quantification of apoptotic cells from (K). (M) Western blot analysis of proliferation marker (PCNA) and apoptosis markers (Bcl‐2, Bax) in HCT116 and SW480 cells transfected with indicated siRNAs. GAPDH was used as a loading control. Data are presented as mean ± SD of three independent experiments. *p < 0.05, **p < 0.01, ***p < 0.001.

Analysis of The Cancer Genome Atlas Colon Adenocarcinoma (TCGA‐COAD) dataset revealed that patients with high DPP7 expression had significantly poorer overall survival (*p* = 0.0001) and progression‐free survival (*p* = 0.0011) compared to those with low DPP7 expression (Figure 1A–C). We examined DPP7 expression in various colorectal cancer (CRC) cell lines and found that HCT116 and SW480 cells expressed notably higher levels of DPP7 (Figure 1D). To investigate the biological function of DPP7 in CRC, we knocked down DPP7 expression in HCT116 and SW480 cells using two specific siRNAs (Figure 1E,F). Both siRNAs effectively reduced DPP7 protein levels. Depletion of DPP7 significantly inhibited cell proliferation, as assessed by the CCK‐8 assay (Figure 1G,H), and colony formation (Figure 1I–L). Additionally, flow cytometry analysis revealed that DPP7 depletion induced apoptosis in both HCT116 and SW480 cells (Figure 1J,K). To validate our functional findings at the molecular level, we examined the expression of key proliferation and apoptosis markers following DPP7 depletion. Western blot analysis revealed that DPP7 depletion significantly reduced PCNA expression in both HCT116 and SW480 cells (Figure 1M), confirming the observed decrease in proliferative capacity. These findings indicate that DPP7 plays a vital role in promoting CRC cell proliferation and survival.

### 3.2 DPP7 Depletion Enhances NK Cell‐Mediated Cytotoxicity Against CRC Cells

**FIGURE 2:**
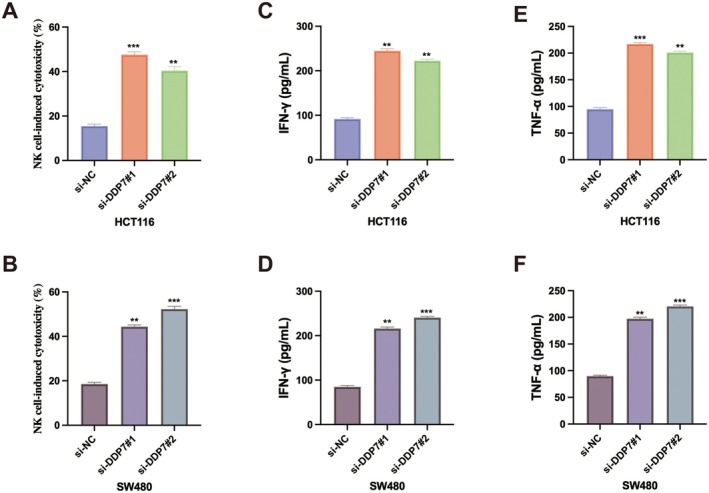
DPP7 depletion enhances NK cell–mediated cytotoxicity against CRC cells. (A‐B) NK cell–induced cytotoxicity against HCT116 (A) and SW480 (B) cells transfected with control siRNA (si‐NC) or DPP7‐specific siRNAs (si‐DPP7#1 and si‐DPP7#2). (C, D) ELISA analysis of IFN‐γ levels in the supernatants of NK cells co‐cultured with HCT116 (C) or SW480 (D) cells transfected with indicated siRNAs. (E, F) ELISA analysis of TNF‐α levels in the supernatants of NK cells co‐cultured with HCT116 (E) or SW480 (F) cells transfected with indicated siRNAs. Data are presented as mean ± SD of three independent experiments. *p < 0.05, **p < 0.01, ***p < 0.001.

Given that tumour immune evasion is a major obstacle in cancer treatment, previous study has reported that DPP7 is associated with NK‐cell activity [19]. We examined whether DPP7 affects the sensitivity of CRC cells to NK cell‐mediated cytotoxicity. NK cell‐induced cytotoxicity assays revealed that DPP7 depletion significantly increased the susceptibility of both HCT116 and SW480 cells to NK cell‐mediated killing (Figure 2A,B). Consistent with this observation, levels of cytokines secreted by NK cells, including IFN‐γ and TNF‐α, were markedly increased when co‐cultured with DPP7‐deficient CRC cells compared to control cells (Figure 2C–F). These results indicate that DPP7 contributes to immune evasion in CRC by impairing NK cell cytotoxicity against tumour cells.

### 3.3 DPP7 Suppresses Disulfidptosis in CRC Cells

**FIGURE 3:**
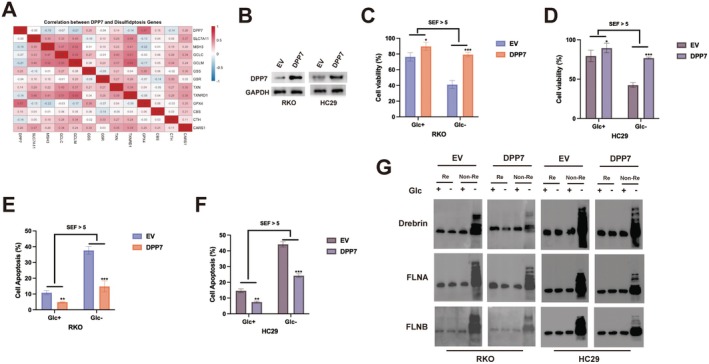
DPP7 suppresses disulfidptosis in CRC cells. (A) Correlation heatmap showing the relationship between DPP7 and disulfidptosis‐related genes. Pearson correlation coefficients are indicated. (B) Western blot analysis of DPP7 protein expression in RKO and HC29 cells transduced with empty vector (EV) or DPP7‐expressing lentivirus. GAPDH was used as a loading control. (C, D) Cell viability of RKO (C) and HC29 (D) cells with or without DPP7 overexpression under glucose‐replete (Glc+) or glucose‐deprived (Glc‐) conditions for 12 h. (E, F) Flow cytometry analysis of apoptosis in RKO (E) and HC29 (F) cells with or without DPP7 overexpression under indicated conditions. (G) Non‐reducing (Non‐Re) and reducing (Re) Western blot analysis of cytoskeletal proteins (Drebrin, FLNA and FLNB) in RKO and HC29 cells with or without DPP7 overexpression under indicated conditions. Data are presented as mean ± SD of three independent experiments. *p < 0.05, **p < 0.01, ***p < 0.001. SEF, starvation effect factor.

Recent studies have identified disulfidptosis as a novel form of regulated cell death characterised by the accumulation of intracellular disulfide bonds [10]. Analysis of the correlation between DPP7 and disulfidptosis‐related genes revealed significant associations (Figure 3A), suggesting a potential role of DPP7 in regulating disulfidptosis. To explore this possibility, we overexpressed DPP7 in RKO and HC29 CRC cell lines that exhibit relatively low endogenous DPP7 expression (Figure 3B). We then subjected cells to glucose deprivation (Glc‐), a known inducer of disulfidptosis. DPP7 overexpression significantly rescued the viability of both RKO and HC29 cells under glucose‐deprived conditions (Figure 3C,D). Furthermore, flow cytometry analysis demonstrated that DPP7 overexpression reduced apoptosis in both cell lines under glucose deprivation (Figure 3E,F). To directly evaluate disulfidptosis, we conducted non‐reducing Western blot analysis of cytoskeletal proteins (Drebrin, FLNA and FLNB), which are known to form disulfide bonds during this process. Under conditions of glucose deprivation, control cells displayed prominent smearing and trailing bands of these proteins, indicating excessive disulfide bond formation. In contrast, DPP7‐overexpressing cells displayed markedly reduced band trailing (Figure 3G), suggesting that DPP7 protects against disulfidptosis during glucose deprivation.

### 3.4 DPP7 Interacts With GPX4 to Regulate Its Protein Stability

**FIGURE 4:**
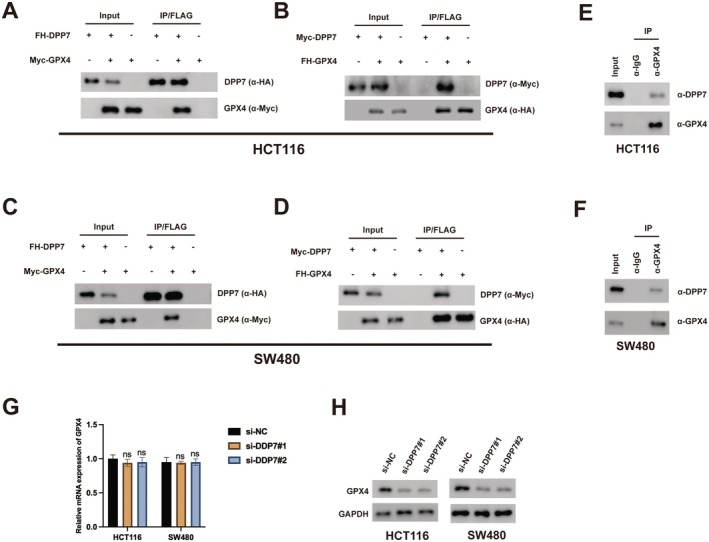
DPP7 interacts with GPX4 to regulate its protein stability. (A–D) Co‐immunoprecipitation (Co‐IP) assays showing the interaction between DPP7 and GPX4 in HCT116 (A, B) and SW480 (C, D) cells. Cells were transfected with FLAG‐HA‐DPP7 (FH‐DPP7) and Myc‐GPX4 or Myc‐DPP7 and FLAG‐HA‐GPX4 (FH‐GPX4) as indicated. Immunoprecipitation was performed using anti‐FLAG antibody, followed by Western blotting with anti‐HA and anti‐Myc antibodies. (E, F) Endogenous interaction between DPP7 and GPX4 in HCT116 (E) and SW480 (F) cells. Cell lysates were subjected to immunoprecipitation with anti‐GPX4 or control IgG antibody, followed by Western blotting with anti‐DPP7 and anti‐GPX4 antibodies. (G) Relative GPX4 mRNA expression in HCT116 and SW480 cells transfected with indicated siRNAs. (H) Western blot analysis of GPX4 protein expression in HCT116 and SW480 cells transfected with indicated siRNAs. GAPDH was used as a loading control. Data are presented as mean ± SD of three independent experiments. ns: not significant.

To uncover the molecular mechanism by which DPP7 suppresses disulfidptosis, we explored potential interactions between DPP7 and known regulators of cellular redox balance. Co‐immunoprecipitation assays revealed that DPP7 physically interacts with glutathione peroxidase 4 (GPX4), a key regulator of cellular redox homeostasis, in both HCT116 and SW480 cells (Figure 4A–F). This interaction was confirmed by reciprocal co‐immunoprecipitation using anti‐GPX4 antibody (Figure 4E,F). Although DPP7 depletion did not affect GPX4 mRNA levels (Figure 4G), it significantly reduced GPX4 protein expression (Figure 4H), suggesting that DPP7 regulates GPX4 at the post‐transcriptional level. These findings indicate that DPP7 may exert its anti‐disulfidptosis function through stabilising GPX4 protein. The fact that DPP7 depletion reduces GPX4 protein levels without affecting mRNA expression suggests post‐transcriptional regulation. Several mechanisms could explain this stabilisation: first, the physical interaction between DPP7 and GPX4 may protect GPX4 from proteasomal or lysosomal degradation by masking recognition sequences for degradation machinery; second, DPP7 binding could enhance GPX4 protein folding stability or prevent misfolding‐induced degradation; third, the interaction might sequester GPX4 in specific cellular compartments where it is protected from degradative processes. Further investigation of protein half‐life, degradation pathways and detailed protein domain interactions will be necessary to fully elucidate the precise mechanism by which DPP7 stabilises GPX4.

### 3.5 Restoration of GPX4 Reverses the Effects of DPP7 Depletion in CRC Cells

**FIGURE 5:**
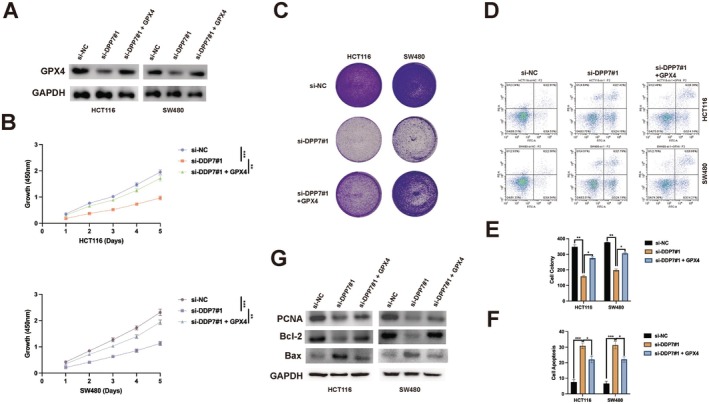
Restoration of GPX4 reverses the effects of DPP7 depletion in CRC cells. (A) Western blot analysis of GPX4 protein expression in HCT116 and SW480 cells transfected with control siRNA (si‐NC), DPP7‐specific siRNA (si‐DPP7#1), or DPP7‐specific siRNA plus GPX4‐expressing plasmid (si‐DPP7#1 + GPX4). GAPDH was used as a loading control. (B) Cell growth curves of HCT116 and SW480 cells transfected as indicated, measured by CCK‐8 assay. (C) Representative images of colony formation assay for HCT116 and SW480 cells transfected as indicated. (D) Quantification of colony numbers from (C). (E) Flow cytometry analysis of apoptosis in HCT116 and SW480 cells transfected as indicated. (F) Quantification of apoptotic cells from (E). (G) Western blot analysis of proliferation marker (PCNA) and apoptosis markers (Bcl‐2, Bax) in HCT116 and SW480 cells transfected with control siRNA (si‐NC), DPP7‐specific siRNA (si‐DPP7#1), or DPP7‐specific siRNA plus GPX4‐expressing plasmid (si‐DPP7#1 + GPX4). GAPDH was used as a loading control. Data are presented as mean ± SD of three independent experiments. *p < 0.05, **p < 0.01, ***p < 0.001.

To determine if GPX4 mediates the tumour‐promoting effects of DPP7, we conducted rescue experiments by overexpressing GPX4 in DPP7‐depletion cells. Western blot analysis confirmed the successful restoration of GPX4 expression in DPP7‐deficient cells (Figure 5A). GPX4 restoration significantly reversed the growth inhibition (Figure 5B,C) and colony formation defects (Figure 5C–E) caused by DPP7 depletion. Additionally, the enhanced apoptosis observed in DPP7‐depletion cells was significantly attenuated by GPX4 overexpression (Figure 5D–F). To further confirm that GPX4 mediates DPP7's effects on cell proliferation and survival, we examined whether GPX4 restoration could reverse the changes in proliferation and apoptosis markers caused by DPP7 depletion. Western blot analysis showed that GPX4 overexpression in DPP7‐depleted cells significantly restored PCNA expression levels (Figure 5G), indicating recovery of proliferative capacity.

**FIGURE 6:**
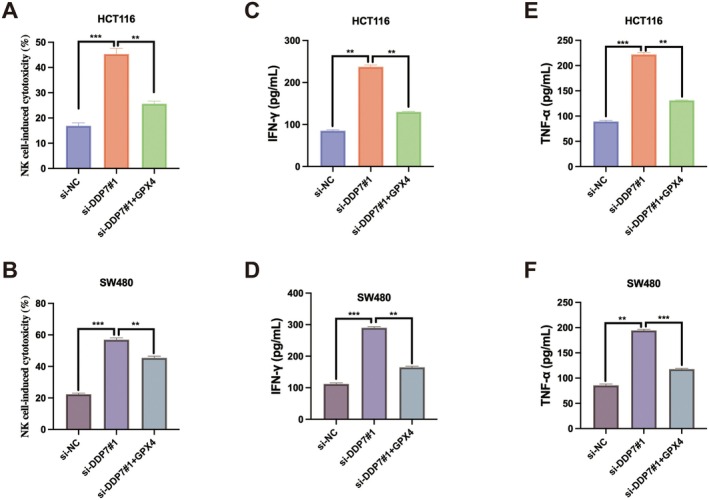
GPX4 mediates DPP7‐induced immune evasion in CRC cells. (A, B) NK cell‐induced cytotoxicity against HCT116 (A) and SW480 (B) cells transfected with control siRNA (si‐NC), DPP7‐specific siRNA (si‐DPP7#1), or DPP7‐specific siRNA plus GPX4‐expressing plasmid (si‐DPP7#1 + GPX4). (C, D) ELISA analysis of IFN‐γ levels in the supernatants of NK cells co‐cultured with HCT116 (C) or SW480 (D) cells transfected as indicated. (E, F) ELISA analysis of TNF‐α levels in the supernatants of NK cells co‐cultured with HCT116 (E) or SW480 (F) cells transfected as indicated. Data are presented as mean ± SD of three independent experiments. *p < 0.05, **p < 0.01, ***p < 0.001.

Furthermore, GPX4 restoration also reduced the sensitivity of DPP7‐depletion cells to NK cell‐mediated cytotoxicity (Figure 6A,B) and decreased the production of IFN‐γ and TNF‐α by co‐cultured NK cells (Figure 6C–F). These results indicate that GPX4 is a key downstream effector of DPP7 in regulating CRC cell growth and immune evasion.

### 3.6 GPX4 Is Required for DPP7‐Mediated Protection Against Disulfidptosis

**FIGURE 7:**
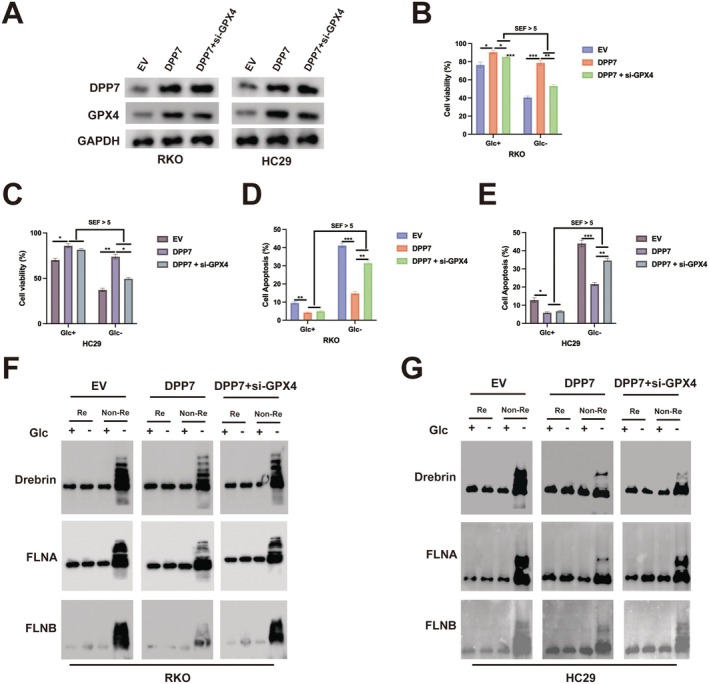
GPX4 is required for DPP7‐mediated protection against disulfidptosis. (A) Western blot analysis of DPP7 and GPX4 protein expression in RKO and HC29 cells transduced with empty vector (EV), DPP7‐expressing lentivirus (DPP7), or DPP7‐expressing lentivirus plus GPX4‐specific siRNA (DPP7 + si‐GPX4). GAPDH was used as a loading control. (B, C) Cell viability of RKO (B) and HC29 (C) cells with indicated genetic modifications under glucose‐replete (Glc+) or glucose‐deprived (Glc‐) conditions for 12 h. (D, E) Flow cytometry analysis of apoptosis in RKO (D) and HC29 (E) cells with indicated genetic modifications under glucose‐replete or glucose‐deprived conditions. (F, G) Non‐reducing (Non‐Re) and reducing (Re) Western blot analysis of cytoskeletal proteins (Drebrin, FLNA and FLNB) in RKO (F) and HC29 (G) cells with indicated genetic modifications under glucose‐replete or glucose‐deprived conditions. Data are presented as mean ± SD of three independent experiments. *p < 0.05, **p < 0.01, ***p < 0.001. SEF, starvation effect factor.

To further validate the role of GPX4 in DPP7‐mediated suppression of disulfidptosis, we knocked down GPX4 in DPP7‐overexpressing RKO and HC29 cells (Figure 7A). While DPP7 overexpression protected cells from glucose deprivation‐induced cell death, GPX4 depletion abolished this protective effect (Figure 7B,C). Similarly, the anti‐apoptotic effect of DPP7 under glucose deprivation was reversed by GPX4 depletion (Figure 7D,E). Non‐reducing Western blot analysis showed that GPX4 depletion reinstated the formation of disulfide bonds in cytoskeletal proteins (Drebrin, FLNA and FLNB) in DPP7‐overexpressing cells under glucose deprivation (Figure 7F,G). These data offer compelling evidence that GPX4 is essential for the DPP7‐mediated protection against disulfidptosis in CRC cells under conditions of metabolic stress.

## 4 Discussion

In this study, we identified DPP7 as a novel regulator of disulfidptosis and immune evasion in colorectal cancer through its interaction with GPX4. Our findings reveal a previously unrecognised molecular mechanism by which CRC cells adapt to metabolic stress and evade immune surveillance, providing potential therapeutic opportunities for colorectal cancer treatment.

DPP7, a member of the dipeptidyl peptidase family, has been primarily studied for its enzymatic activity in protein degradation and peptide processing [20, 21]. However, its role in cancer biology, particularly in colorectal cancer, has remained largely unexplored. Our analysis of the TCGA‐COAD dataset showed that DPP7 is significantly overexpressed in colorectal cancer tissues compared to adjacent normal tissues, and its high expression is associated with poor overall and progression‐free survival. These clinical data suggest that DPP7 may serve as a potential prognostic biomarker for CRC patients. Functional studies further revealed that DPP7 promotes CRC cell proliferation and survival, as evidenced by reduced cell growth and increased apoptosis following DPP7 depletion in HCT116 and SW480, two CRC cell lines with high endogenous DPP7 expression.

A particularly intriguing aspect of our study is the role of DPP7 in regulating NK cell‐mediated anti‐tumour immunity. NK cells are critical components of the innate immune system that recognise and eliminate malignant cells through direct cytotoxicity and cytokine production [22, 23]. Our results showed that DPP7 depletion significantly enhanced the susceptibility of CRC cells to NK cell–mediated killing and increased the production of IFN‐γ and TNF‐α by co‐cultured NK cells. These findings suggest that DPP7 contributes to immune evasion in colorectal cancer, potentially by modulating tumour cell susceptibility to NK cell recognition and/or attack. Previous studies have implicated several mechanisms in tumour immune evasion, including downregulation of stress‐induced ligands for NK cell receptors, secretion of immunosuppressive factors and alterations in metabolic pathways [24, 25, 26]. Further investigations are needed to clarify the precise mechanism by which DPP7 regulates the interaction between CRC cells and NK cells.

Disulfidptosis, characterised by the accumulation of intracellular disulfide bonds and protein aggregation, has emerged as a novel form of regulated cell death implicated in various pathological conditions [10, 27, 28]. Our study provides the first evidence linking DPP7 to the regulation of disulfidptosis in cancer cells. We found that DPP7 overexpression protected RKO and HC29 cells, which express relatively low endogenous DPP7 levels, from glucose deprivation‐induced disulfidptosis. This protective effect was evidenced by increased cell viability, reduced apoptosis and decreased formation of disulfide bonds in cytoskeletal proteins (Drebrin, FLNA and FLNB) under conditions of glucose deprivation. These findings suggest that DPP7 may confer a survival advantage to CRC cells under metabolic stress, a common feature of the tumour microenvironment.

Mechanistically, we identified GPX4 as a critical mediator of DPP7's functions in CRC cells. GPX4, a glutathione peroxidase that catalyses the reduction of lipid hydroperoxides, is essential for maintaining redox homeostasis and preventing ferroptosis, another form of regulated cell death [11, 29]. Our co‐immunoprecipitation assays revealed a physical interaction between DPP7 and GPX4 in CRC cells. Notably, DPP7 depletion decreased GPX4 protein expression without altering its mRNA levels, indicating that DPP7 regulates GPX4 at the post‐transcriptional level, potentially by enhancing its protein stability. This finding adds a new layer to our understanding of GPX4 regulation, which has previously been shown to be modulated by transcriptional, post‐transcriptional and post‐translational mechanisms [30, 31, 32].

The mechanism by which DPP7 stabilises GPX4 likely involves direct interference with protein degradation pathways. The physical interaction between DPP7 and GPX4, combined with the post‐transcriptional nature of GPX4 regulation (decreased protein without mRNA changes), suggests that DPP7 protects GPX4 from degradative processes. This could occur through several potential mechanisms: masking of ubiquitination sites that target GPX4 for proteasomal degradation, sequestration in cellular compartments away from degradative machinery, or recruitment of stabilising cofactors. Future studies involving protein half‐life analysis, ubiquitination status examination and proteasomal degradation pathway investigation will be essential to fully elucidate the precise molecular mechanism underlying this important regulatory interaction.

The functional significance of the DPP7‐GPX4 interaction was further confirmed through rescue experiments. Reintroducing GPX4 expression in DPP7‐depletion cells reversed the growth inhibition, reduced apoptosis and diminished the increased sensitivity to NK cell–mediated cytotoxicity induced by DPP7 depletion. Conversely, GPX4 depletion abolished the protective effect of DPP7 overexpression against glucose deprivation–induced disulfidptosis. These results establish GPX4 as a downstream effector of DPP7 in regulating both CRC cell survival and immune evasion.

Our study bridges two seemingly distinct processes—disulfidptosis and immune evasion—through the DPP7‐GPX4 axis. This connection is particularly significant given the emerging evidence linking cellular stress responses to immune recognition and elimination of cancer cells [33]. Cellular stress, including oxidative stress and metabolic perturbations, can induce the expression of ligands for NK cell activating receptors, thereby enhancing tumour cell recognition by NK cells [34]. By regulating GPX4‐dependent redox homeostasis, DPP7 may impact the expression of stress‐induced ligands, thereby influencing NK cell‐mediated immune surveillance. Future studies should investigate this potential mechanism and its implications for cancer immunotherapy.

The identification of the DPP7‐GPX4 axis in regulating disulfidptosis and immune evasion has potential therapeutic implications. Targeting DPP7 and/or its interaction with GPX4 may represent a novel strategy to enhance the efficacy of immunotherapy in colorectal cancer. Several approaches could be explored, including the development of specific inhibitors against DPP7's enzymatic activity or disruptors of the DPP7‐GPX4 interaction. Additionally, combining these targeted therapies with immune checkpoint inhibitors or adoptive NK cell transfer might provide synergistic anti‐tumour effects by simultaneously inhibiting intrinsic survival mechanisms and enhancing immune‐mediated tumour cell elimination.

There are several limitations to our study that should be addressed in future investigations. First, the precise molecular mechanism by which DPP7 regulates GPX4 protein stability remains to be elucidated. Second, the potential involvement of other DPP family members, particularly DPP4, which has been implicated in cancer progression and immune regulation [35], should be explored to determine whether they function redundantly or distinctly from DPP7 in colorectal cancer. Third, the relevance of our findings to other cancer types and their potential clinical applications warrant further evaluation in diverse cancer models and patient cohorts. While we have established that DPP7 stabilises GPX4 through protein–protein interaction, our study does not fully elucidate the precise molecular mechanism underlying this stabilisation. The specific degradation pathways that are inhibited, the role of post‐translational modifications, and the detailed structural basis of the interaction remain to be characterised. Additionally, whether this stabilisation occurs in specific subcellular compartments or involves additional cofactors requires further investigation.

In conclusion, our study identifies DPP7 as a novel regulator of disulfidptosis and immune evasion in colorectal cancer through its interaction with GPX4. By enhancing GPX4 protein stability, DPP7 shields CRC cells from disulfidptosis under metabolic stress and decreases their vulnerability to NK cell–mediated cytotoxicity. These findings not only deepen our understanding of the molecular mechanisms driving colorectal cancer progression but also identify potential therapeutic targets for improving CRC treatment outcomes. Future studies should focus on further characterising the DPP7‐GPX4 axis and exploring its therapeutic potential in colorectal cancer and possibly other malignancies.

## Author Contributions

**Ruibing Li:** data curation (equal), formal analysis (equal), investigation (equal), software (equal), validation (equal), visualization (equal). **Xinyou Wang:** data curation (equal), formal analysis (equal), validation (equal), visualization (equal). **Jun Liu:** formal analysis (equal), investigation (equal), methodology (equal). **Zeyu Cai:** software (equal), visualization (equal). **Zhu Li:** investigation (equal), project administration (equal), supervision (equal). **Qiang Tao:** resources (equal), supervision (equal), writing – original draft (equal). **Chong Wang:** conceptualization (equal), funding acquisition (equal), methodology (equal), project administration (equal), supervision (equal), writing – original draft (equal).

## Conflicts of Interest

The authors declare no conflicts of interest.

## Data Availability

The data that support the findings of this study are available from the corresponding author upon reasonable request.
